# Effect of polypharmacy on plasma bepridil concentration in patients with heart failure: a multicenter retrospective study

**DOI:** 10.1186/s40780-023-00278-x

**Published:** 2023-03-06

**Authors:** Yuki Asai, Hiroki Arihara, Saki Omote, Ena Tanio, Saena Yamashita, Takashi Higuchi, Ei Hashimoto, Momoko Yamada, Hinako Tsuji, Yoshihiro Kondo, Makoto Hayashi, Yoshiaki Yamamoto

**Affiliations:** 1 Pharmacy, National Hospital Organization Mie Chuo Medical Center, 2158-5 Hisaimyojin, Tsu, Mie 514-1101 Japan; 2 Pharmacy, National Hospital Organization Kanazawa Medical Center, 1-1, Shimoishibiki, Kanazawa, Ishikawa 920-0850 Japan; 3 Pharmacy, National Hospital Organization Nagoya Medical Center, 4-1-1, Sannomaru, Naka-ku, Nagoya, 460-0001 Japan; 4 Department of Clinical Research, National Hospital Organization Shizuoka Institute of Epilepsy and Neurological Disorders, 886 Urushiyama, Shizuoka, 420-8688 Japan

**Keywords:** Bepridil, Heart failure, Polypharmacy, Drug-drug interaction, Therapeutic drug monitoring

## Abstract

**Background:**

Polypharmacy, defined as the concurrent use of over six drugs, is common in the treatment of heart failure (HF); however, unpredictable drug interactions with bepridil may occur. In this study, we have elucidated the influence of polypharmacy on plasma bepridil concentrations in patients with HF.

**Methods:**

We conducted a multicenter retrospective study involving 359 adult patients with HF who received oral bepridil. Because QT prolongation is an adverse effect following plasma bepridil concentrations ≥800 ng/mL, the risk factors for patients achieving these concentrations at steady state were elucidated via multivariate logistic regression. The correlation between bepridil dose and plasma concentration was examined. The effect of polypharmacy on the value of the concentration-to-dose (C/D) ratio was investigated.

**Results:**

A significant relationship was observed between bepridil dose and plasma concentration (*p* <  0.001), and the intensity of the correlation was moderate (*r* = 0.503). Based on multivariate logistic regression, the adjusted odds ratios for a daily dose of bepridil ≥1.6 mg/kg, polypharmacy, and concomitant of aprindine, a cytochrome P450 2D6 inhibitor, were 6.82 (95% coefficient interval: 2.104–22.132, *p* = 0.001), 2.96 (95% coefficient interval: 1.014–8.643, *p* = 0.047), and 8.63 (95% coefficient interval: 1.684–44.215, *p* = 0.010), respectively. Despite the moderate correlation in non-polypharmacy, the correlation was not observed in polypharmacy. Therefore, inhibiting metabolism, along with other mechanisms, may contribute to the polypharmacy-induced increase in plasma bepridil concentrations. Moreover, the C/D ratios in the groups receiving 6–9 and 10≤ concomitant drugs were 1.28- and 1.70-fold higher than in those receiving <6 drugs, respectively.

**Conclusions:**

Plasma bepridil concentrations may be influenced by polypharmacy. Moreover, the plasma bepridil concentration increased in correlation with the number of concomitant drugs used. Although the mechanism of this increase could not be determined, plasma bepridil concentrations should be periodically monitored for safe use in patients with HF.

**Trial registration:**

Retrospectively registered.

## Background

Bepridil has various channel-blocking properties and has been widely used as an antiarrhythmic drug [1]. Recently, the use of bepridil administration has been considered a second-line therapy for patients with atrial fibrillation (AF) who are refractory to treatment with other antiarrhythmic drugs in Japan [2–4]. However, bepridil treatment often causes QT prolongation and torsades de pointes [3]. Matsui et al. reported that the QTc interval is strongly associated with plasma bepridil concentration [5], and it is associated with an increased risk when the plasma concentration of bepridil exceeds 800 ng/mL [3], indicating that therapeutic drug monitoring of plasma bepridil concentration is essential for safety in clinical settings.

Bepridil treatment is likely to cause inter-individual variability in plasma concentrations owing to its low bioavailability (< 60%), large volume of distribution (8 L/kg), and long half-life (48–80 h) [6]. In patients with heart failure (HF), the clearance of various antiarrhythmic drugs has been reported to decrease owing to a large change in blood flow in the systemic circulation [7], suggesting that the dose adjustment of bepridil in patients with HF, who represent a unique population, is more important than that in patients without HF. However, the factors influencing the increase in plasma bepridil concentration in patients with HF remain unknown.

As patients with HF have several comorbidities, drug therapy for HF currently involves polypharmacy [8]. In Japan, polypharmacy is generally defined as the concomitant use of six or more drugs due to the increased risk of side effects in patients taking six and more than six drugs concomitantly [9, 10]. Typically, polypharmacy increases the risk of potential and unpredictable drug–drug interactions associated with drug-metabolizing enzymes and efflux transporters, such as Cytochrome P450 (CYP) and P-glycoprotein (P-gp) [11]. This phenomenon causes an increase or decrease in the blood concentrations of concomitant drugs. In addition, a previous study revealed that bepridil is mainly metabolized by hepatic CYP2D6 and partly by CYP3A [12]. Moreover, bepridil is a substrate of P-gp [13], suggesting that polypharmacy in HF may alter plasma bepridil concentrations; however, the details have not been elucidated. Therefore, a multicenter retrospective study was performed to clarify the effect of polypharmacy on plasma bepridil concentrations in patients with HF.

## Methods

### Study design

This multicenter retrospective study was conducted at three hospitals belonging to the Tokai-Hokuriku Group of the National Hospital Organization (Kanazawa Medical Center, Mie Chuo Medical Center, and Nagoya Medical Center).

### Participants

The data on patients receiving oral bepridil at the three hospitals from January 1, 2011, to December 31, 2021, were collected. In this study, patients with HF were defined as those diagnosed with HF by an attending physician based on a comprehensive evaluation of their symptoms and medical history. The inclusion criteria were defined as HF patients of age ≥ 18 years who were receiving oral bepridil. The following patients were excluded from the analysis as follows: (1) patients with no plasma bepridil measurement, (2) patients without echocardiography data, (3) patients undergoing hemodialysis, and (4) missing data. In this study, patients who had been administered bepridil for at least 28 days beyond the steady state were selected for analysis as previously reported [14]. Blood samples were stored at 4 °C until measurement. The measurement of their plasma bepridil concentrations via liquid chromatography-tandem mass spectrometry was outsourced to SRL, Inc. (Tokyo, Japan) or LSI Medience Corporation (Tokyo, Japan). In addition, patients with bepridil concentration below the limit of quantification (< 20 ng/mL) were excluded. For patients who received therapeutic drug monitoring of bepridil twice or more during this study period, drug concentration data obtained from the last measurement were included in the analysis.

### Data collection and analysis

The collection time of the plasma sample included data from before administration of bepridil to up to 6 h after administration. To assess risk factors for achieving plasma bepridil concentrations ≥800 ng/mL at steady state, the eligible patients were divided into two groups based on their bepridil concentrations: ≥800 ng/mL and < 800 ng/mL.

The C/D ratio was calculated using the following equation:

C/D ratio of bepridil = plasma concentration of bepridil (ng/mL) / dose of bepridil (mg/day/kg body weight).

In this study, we defined the polypharmacy group as those who use six or more drugs, whereas the non-polypharmacy group was those who took fewer than six drugs. The relationship between plasma bepridil concentrations ≥800 ng/mL and baseline characteristics, including sex, age, height, body weight, body mass index, serum creatinine, creatinine clearance (Ccr), number of concomitant drugs used, typical inducers of CYPs (phenytoin, carbamazepine, phenobarbital, and rifampicin) [15], typical inhibitors of CYPs (erythromycin, clarithromycin, protease inhibitors, and azole antifungals) [15], aprindine, a competitive inhibitor of CYP2D6 [12], typical inhibitor of P-gp (amiodarone, diltiazem, nicardipine, nifedipine, propranolol, quinidine, cyclosporin, and tacrolimus) [16–18], and left ventricular ejection fraction (LVEF), were examined. LVEF was measured using echocardiographic equipment provided at each hospital. Ccr was estimated using the Cockcroft–Gault formula [19].

The patient’s medical history and duration of bepridil treatment were collected from medical records.

### Statistical analysis

Correlations between bepridil dosage and its plasma concentration were analyzed using Spearman correlation coefficient analysis. Univariate analyses were performed as follows for continuous and categorical variables, respectively. Differences in continuous variables between the ≥800 ng/mL and < 800 ng/mL groups were analyzed using the Mann–Whitney U test as they followed a non-normal distribution. Categorical variables were compared using the chi-square test. Fisher’s exact test was selected to include one cell with an expected value of < 5 on a 2 × 2 contingency table. In the multivariate logistic regression analysis, the objective variable constituted plasma bepridil concentrations ≥800 ng/mL, whereas the explanatory variables included age, which reportedly influences plasma bepridil concentration [20], and factors that showed *p* <  0.05 in the univariate analysis. For enhanced clarity of clinical settings, when the continuous variables were included in the multivariate logistic regression model, the continuous variables were converted to categorical variables based on specified cut-off values. Specifically, the cut-off values for age and daily bepridil dose were the median values obtained considering all the eligible patients (Table 1). For Ccr, the cutoff value was Ccr ≤ 30 mL/min, which signifies severe renal impairment, and for LVEF, it was LVEF ≤50%, which indicates a reduced LVEF. Furthermore, the Hosmer–Lemeshow test was used to assess the goodness of fit of the multivariate logistic regression model (*p* > 0.05 was considered statistically significant). Multicollinearity was also evaluated using the variance inflation factor (VIF). To determine the number of concomitant drugs influencing the C/D ratio, the three groups were compared using the Kruskal–Wallis test, followed by Bonferroni correction for comparisons between groups. Statistical analyses were performed using SPSS Statistics version 27 (IBM Japan, Tokyo, Japan), and the significance level was set at *p* <  0.05.

**Table 1:** Summary of patient data

Factors	*n* = 359
Sex (Male/Female)	238/121
Age (years)	71 (64, 79)^d^
Height (m)	1.64 (1.55, 1.71)^d^
Body weight (kg)	62.55 (54.68, 72.40)^d^
Body mass index (kg/m^2^)	23.41 (21.15, 25.65)^d^
Serum creatinine (mg/dL)	0.88 (0.74, 1.03)^d^
Ccr (mL/min)	64.63 (47.56, 86.03)^d^
Daily dose of bepridil (mg/kg body weight)	1.58 (1.24, 2.02)^d^
Period of bepridil treatment (day)	356 (124, 1404)^d^
Plasma bepridil concentration (ng/mL)	300 (157, 491)^d^
C/D ratio of bepridil (ng/mL) / (mg/day/kg)	186 (108, 278)
LVEF (%)	65.1 (59.1, 69.9)^d^
HFrEF^a^, n (%)	14 (3.9)
HFmrEF^b^, n (%)	28 (7.8)
HFpEF^c^, n (%)	317 (88.3)
Patient’s medical history, n (%)	
Coronary artery bypass graft	3 (0.8)
Graft replacement	1 (0.3)
Atrial fibrillation	346 (94.3)
Heart valve replacement or formation	8 (2.2)
Concomitant drugs for HF and comorbidities, n (%)	
ACE inhibitor/ARB	116 (32.3)
*β*-blocker	218 (60.7)
Calcium-channel blocker	98 (27.3)
Statins	111 (30.9)
Diuretics	89 (24.8)
Antidiabetic drugs	40 (11.1)
Anticoagulant drugs	286 (79.7)

*ACE* Angiotensin converting enzyme, *ARB* Angiotensin II receptor blocker, *Ccr* Creatinine clearance, *C/D* Concentration-to-dose, *HFmrEF* Heart failure with mid-range ejection fraction; *HFpEF* Heart failure with preserved ejection fraction, *HFrEF* Heart failure with reduced ejection fraction, *LVEF* Left ventricular ejection fraction

^a^LVEF< 40%

^b^40%≦LVEF< 50%

^C^50%≦LVEF

^d^Each value represents the median (25 to 75% percentile)

## Results

### Patients

**Fig. 1:**
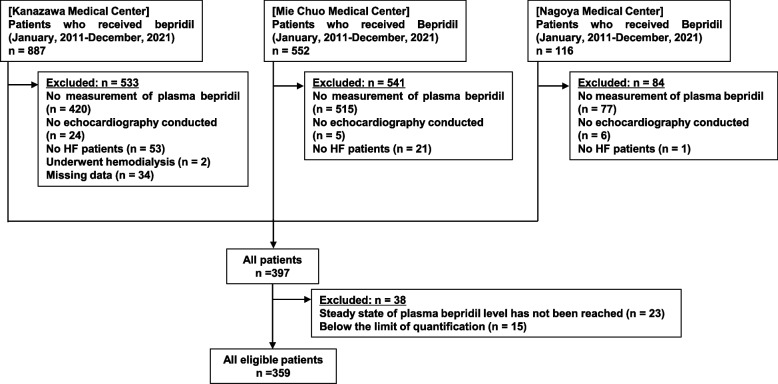
Flowchart for the selection of included patients

In total, 359 patients with HF who received oral bepridil were eligible for analysis (Fig. 1). The characteristics of the patients are listed in Table 1. A total of 359 patients [238 men, 121 women; median age: 71 years (range: 64–79 years); body mass index: 23.41 kg/m^2^ (range: 21.15–25.65 kg/m^2^)] were included. The median dose of bepridil was 1.58 mg/day/kg, and the median treatment period was nearly 1 year. Approximately 90% of the patients were diagnosed with HF with preserved ejection fraction, and less than 5% had HF with reduced ejection fraction. The patients with HF enrolled in this study had AF as a comorbidity. The value of serum albumin could not be investigated since there were many cases with missing data about serum albumin levels (65%, *n* = 235/359).

### Relationship between bepridil dose and plasma bepridil concentration

**Fig. 2:**
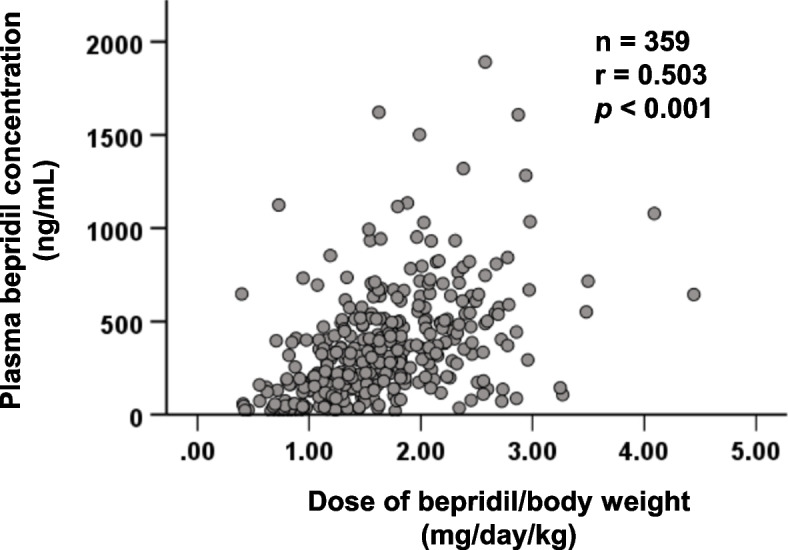
Correlation between plasma bepridil concentration and its daily dose in patients with HF

**Fig. 3:**
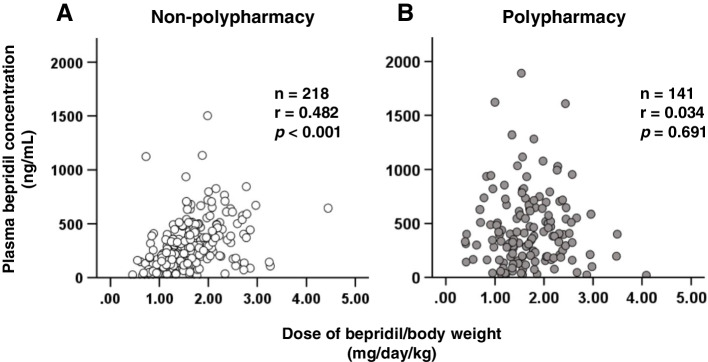
Correlation between bepridil daily dose and plasma concentration under non-polypharmacy and polypharmacy in patients with HF. **A** Non-polypharmacy (**B**) Polypharmacy

A significant moderate correlation was observed between the bepridil dose and its plasma concentration (*r* = 0.503, *p* <0.001) (Fig. 2). A significant correlation was observed between the bepridil dose and its plasma concentration in the non-polypharmacy group (Fig. 3A), whereas no correlation was observed in the polypharmacy group (Fig. 3B).

### Factors contributing to plasma bepridil concentrations ≥800 ng/mL

Univariate analysis revealed that age (*p* = 0.019), daily dose of bepridil (*p* < 0.001), serum creatinine (*p* = 0.027), Ccr (*p* = 0.002), number of concomitant drugs (*p* <0.001), LVEF (*p* = 0.029), and concomitant use of aprindine (*p* = 0.023) all contributed to plasma bepridil concentrations reaching ≥800 ng/mL in patients (Table 2). Multivariate logistic regression analysis revealed that the adjusted odds ratios for a daily dose of bepridil ≥1.6 mg/kg, polypharmacy, and concomitant of aprindine were 6.82 (95% coefficient interval: 2.104–22.132, *p* = 0.001), 2.96 (95% coefficient interval: 1.014–8.643, *p* = 0.047), and 8.63 (95% coefficient interval: 1.684–44.215, *p* = 0.010), respectively (Table 3). The Hosmer–Lemeshow test determined the *p*-value for the goodness of fit of the multivariate logistic regression to be 0.366, and the accuracy rate of the regression was 93.9%. The VIF values for age, daily bepridil dose, Ccr, LVEF, and number of concomitant drugs were 2.339, 1.042, 2.416, 1.063, and 1.204, respectively.

**Table 2:** Univariate analysis of factors contributing to plasma bepridil concentration in patients with HF

Factors	<800 ng/mL	≥800 ng/mL	*p* value
	*n* = 335	*n* = 24	
Sex (Male/Female)	223/112	15/9	0.684^a^
Age	70.33 ± 10.89^d^	75.08 ± 10.51^d^	0.019^c^
Height (m)	1.63 ± 0.10^d^	1.59 ± 0.11^d^	0.058^c^
Body weight (kg)	63.45 ± 13.81^d^	58.21 ± 12.17^d^	0.079^c^
Body mass index (kg/m^2^)	23.63 ± 3.79^d^	22.98 ± 3.87^d^	0.570^c^
Daily dose of bepridil (mg/kg body weight)	1.61 ± 0.59^d^	2.18 ± 0.69^d^	<0.001^c^
Serum creatinine (mg/dL)	0.93 ± 0.41^d^	1.12 ± 0.47^d^	0.027^c^
Ccr (mL/min)	69.68 ± 29.43^d^	52.12 ± 28.43^d^	0.002^c^
LVEF (%)	63.51 ± 10.65^d^	59.52 ± 10.23^d^	0.029^c^
Number of concomitant drugs	4.70 ± 3.51^d^	8.17 ± 3.87^d^	<0.001^c^
Properties of concomitant drugs			
Typical inducer of CYP enzymes			
Carbamazepine, n (%)	1 (0.30)	0 (0)	1.000^b^
Typical inhibitor of CYP enzymes			
Clarithromycin, n (%)	1 (0.30)	0 (0)	1.000^b^
CYP2D6 inhibitor			
Aprindine, n (%)	7 (2.1)	3 (14.3)	0.023^b^
Typical inhibitor of P-gp			
Verapamil	4 (1.2)	1 (4.3)	0.294^b^
Digoxin	4 (1.2)	0 (0.0)	1.000^b^
Amiodarone	3 (0.9)	0 (0.0)	1.000^b^
Diltiazem	6 (1.8)	0 (0.0)	1.000^b^
Nifedipine	5 (1.5)	0 (0.0)	1.000^b^

*Ccr* Creatinine clearance, *CYP* Cytochrome P450, *P-gp* P-glycoprotein, *LVEF* Left ventricular ejection fraction

^a^Chi-square test. ^b^Fisher’s exact test. ^c^Mann–Whitney U test. ^d^Each value represents the mean ± standard deviation

**Table 3:** Factors influencing the plasma bepridil concentration ≥800 ng/mL in patients with HF on multivariate logistic analysis

Factors	Adjusted OR	95% CI	*p* value
Age ≥70 years	1.90	0.571–6.321	0.295
Daily dose of bepridil ≥1.6 mg/kg body weight	6.82	2.104–22.132	0.001
Polypharmacy	2.96	1.014–8.643	0.047
Concomitant of aprindine	8.63	1.684–44.215	0.010
Ccr ≤30 mL/min	2.75	0.848–8.927	0.092
LVEF ≤50%	1.76	0.578–5.358	0.320

*Ccr* Creatinine clearance, *LVEF* Left ventricular ejection fraction, *OR* Odds ratio, *95% CI* 95% coefficient interval

### Association between polypharmacy and plasma bepridil concentration

**Fig. 4:**
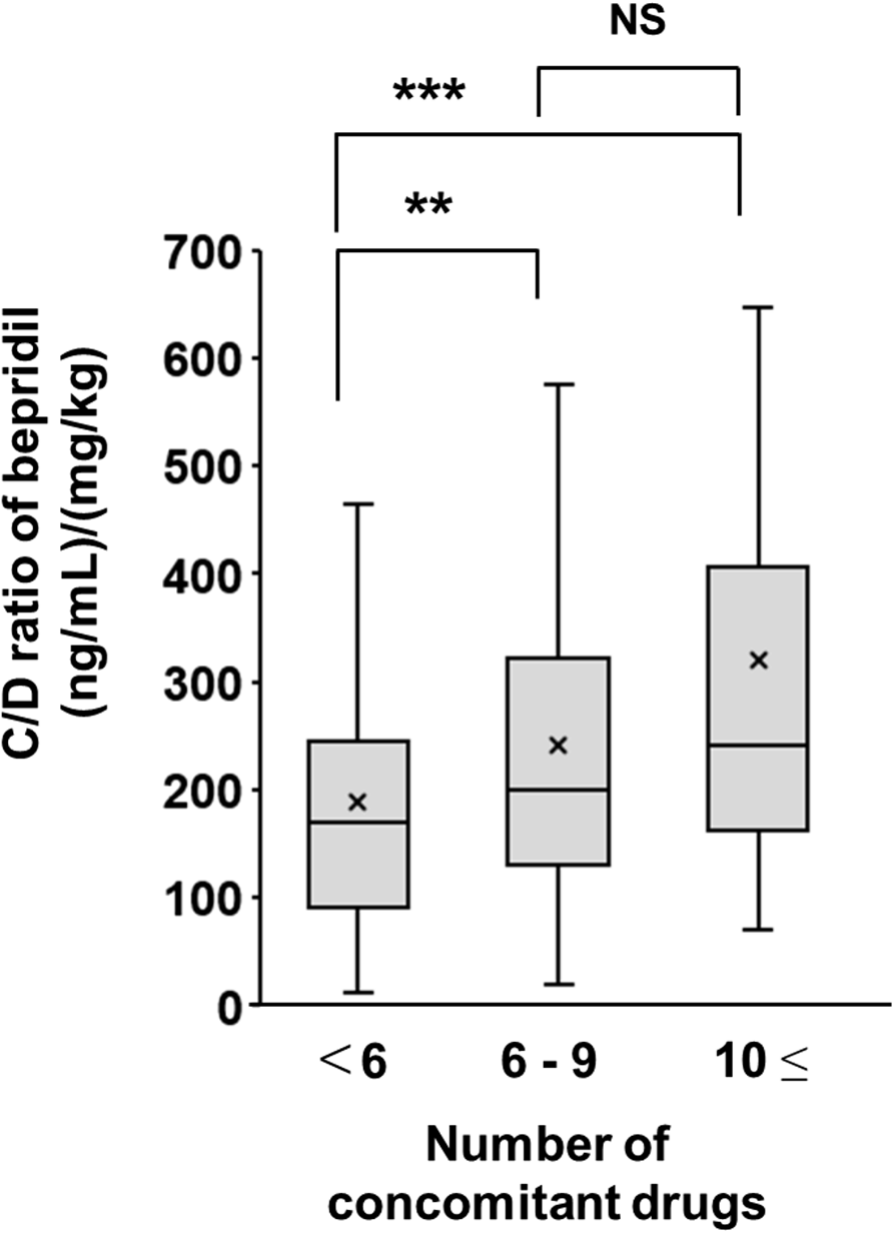
Correlation of the number of concomitant drugs used with the C/D ratio of bepridil in patients with HF. Each column represents the mean ± standard deviation. **p < 0.01 and ***p < 0.001 compared with the number of concomitant drugs < 6. NS, not significant; C/D, concentration-to-dose ratio

Although the C/D ratios in the groups receiving 6–9 and 10≤ drugs were 1.28- and 1.70-fold higher than that in the group receiving < 6 drugs, respectively, no significant difference was observed in C/D ratios between the groups receiving 6–9 and 10≤ drugs (Fig. 4).

## Discussion

As the condition of HF worsens, the number of concomitant drugs required to control the disease rises [8], resulting in drug interactions and may affect the pharmacological effect of bepridil. Therefore, it is necessary to examine the effect of the increase in the number of concomitant drugs on plasma bepridil concentrations. To the best of our knowledge, this is the first study to demonstrate that polypharmacy may increase the plasma bepridil concentration in patients with HF.

Although the rate of AF conversion to sinus rhythm is associated with a linear dose-response relationship of bepridil [21], excessive plasma bepridil concentration causes QT prolongation and torsades de pointes. A meta-analysis revealed that QT prolongation is associated with cardiovascular and sudden death [22], suggesting that patients with HF should be carefully monitored. Several studies reported that plasma bepridil concentration correlates with the daily dose [5, 14], and this phenomenon is consistent with the findings of the present study (Fig. 2). However, excessively high plasma concentrations of bepridil were observed in a few cases. Metabolic saturation of bepridil mediated by CYP2D6 was observed; the CYP2D6*10 allele may reduce oral clearance [12]. Moreover, patients with HF experience major fluctuations in systemic circulation and organ perfusion as HF progresses [23], suggesting that intra-individual variability in the pharmacokinetics of bepridil may be more significant in patients with HF than that in other patients. Therefore, it was considered necessary to conduct a population pharmacokinetic analysis to take into account intra- and inter-individual variability in plasma bepridil concentration in HF patients for further investigation.

The excretion rate of unchanged bepridil in urine is < 0.1%, and dose adjustment is not considered essential in end-stage renal disease [24]. Therefore, a decrease in Ccr is unlikely to induce plasma bepridil concentrations ≥800 ng/mL. Shimamoto et al. reported that the clearance of vancomycin, a typical drug excreted into the urine, is affected by LVEF reduction in congestive HF [25]. As a mechanism for this alteration, they speculated that the renal blood flow reduces owing to decreased cardiac output. Thus, the lack of any effect of LVEF ≤50% on plasma bepridil concentration could be attributed to its excretion pathway. Decreased cardiac output also significantly reduces hepatic blood flow in patients with HF [23]. Furthermore, an animal study revealed that HF-induced hepatic ischemia reduces the clearance of propranolol, a substrate of CYP2D [26], suggesting that the decline in hepatic blood flow mediated by LVEF decrease may require further examination for alterations in the CYP2D6-induced metabolic activity of bepridil. However, as only 3.9% of the patients with HF enrolled in the present study had an LVEF of <40% (Table 1), the effect of LVEF attenuation on bepridil clearance was likely under-assessed.

A previous study revealed that human serum albumin may have at least two bepridil binding sites [27]. Although an in vitro study found that the free fraction of bepridil increased following the addition of verapamil, nifedipine, diltiazem, disopyramide, or warfarin to a patient’s treatment regimen, these added concentrations were much higher than the clinical blood concentration [27]. Therefore, it is unlikely that competitive inhibition of serum albumin binding between bepridil and other drugs would occur in clinical settings. Although the serum albumin levels of enrolled patients were not available, the concomitant rates of verapamil, nifedipine, and diltiazem were not significantly different between the ≥800 ng/mL and <800 ng/mL serum concentration groups (Table 2), suggesting that variations in the protein binding rate of bepridil have a very low impact on the results of this study.

Concomitant use of aprindine, a competitive inhibitor of CYP2D6, maintains the dose–blood concentration correlation; however, it increases its slope compared to that without aprindine. The concomitant use of aprindine was also shown in this study to be a risk factor for increasing plasma levels of bepridil (Table 3). CYP2D6 inhibition was considered one of the mechanisms affecting plasma concentrations of bepridil, and a correlation analysis was performed between dose and plasma concentration under polypharmacy status. However, while a moderate correlation for non-polypharmacy was observed (Fig. 3A), a correlation was not observed for polypharmacy (Fig. 3B). Bepridil is a substrate of P-gp, an efflux transporter, and its absorption in the small intestine may be affected [13]. Although typical inhibitors of P-gp would not affect plasma bepridil concentration in our results (Table 2), it was possible that the P-gp inhibitory effect could not be fully evaluated due to the very small number of patients taking these drugs. Therefore, it was speculated that polypharmacy could have influenced the absorption, metabolism, or other pathways associated with the pharmacokinetics of bepridil; however, the details remain unknown. HF progression is slow; the number of therapeutic drugs used increases with comorbidities [28]. The C/D ratio increases in correlation with an increase in the number of concomitant drugs used (Fig. 4), indicating that there may be a risk of dose-independent increases in the plasma concentration of bepridil as HF progresses.

In the multivariate logistic regression model, the number size corresponding to bepridil concentrations ≥800 ng/mL group was 24, which likely resulted in overfitting owing to 6 explanatory variables. However, the *p* value obtained in the Hosmer–Lemeshow test was 0.366, and the accuracy was 93.9%, suggesting that this model may show acceptable fit. In addition, the low VIF value suggested the absence of multicollinearity.

Our study had several limitations. First, the blood collection time included non-trough sampling. However, steady-state plasma bepridil concentration did not differ between 2 and 10 h after bepridil treatment [29] owing to its long half-life (48–80 h), suggesting that the time of blood collection had a minor effect on plasma bepridil concentrations. Second, because this study design was a retrospective observation, the effect of the edema, ascites, and urine volume on plasma bepridil concentrations could not be evaluated. Third, the inclusion of patients with poor adherence to bepridil would result in underestimating the plasma drug levels. Considering these limitations, further prospective studies are needed to be conducted. Nonetheless, the vital aspects of this study remain that it was a multicenter experiment with enough eligible patients with HF and minimal enough bias to allow for scientific rigor and external validity.

## Conclusions

Plasma concentrations of bepridil may be influenced by polypharmacy. Because the plasma bepridil concentration further increased in correlation with the number of concomitant drugs used, periodic monitoring of plasma bepridil concentration should be performed to facilitate safe use in patients with HF.

## Data Availability

The data supporting the findings of this study are available from the corresponding author upon reasonable request.
